# Effects of Highly Conserved Major Histocompatibility Complex (MHC) Extended Haplotypes on Iron and Low CD8^+^ T Lymphocyte Phenotypes in *HFE* C282Y Homozygous Hemochromatosis Patients from Three Geographically Distant Areas

**DOI:** 10.1371/journal.pone.0079990

**Published:** 2013-11-25

**Authors:** Mónica Costa, Eugénia Cruz, James C. Barton, Ketil Thorstensen, Sandra Morais, Berta M. da Silva, Jorge P. Pinto, Cristina P. Vieira, Jorge Vieira, Ronald T. Acton, Graça Porto

**Affiliations:** 1 Basic and Clinical Research on Iron Biology (BCRIB), Institute for Molecular and Cell Biology (IBMC), University of Porto, Porto, Portugal; 2 Clinical Hematology, Santo António Hospital – Centro Hospitalar do Porto, Porto, Portugal; 3 Southern Iron Disorders Center and Department of Medicine, University of Alabama at Birmingham, Birmingham, Alabama, United States of America; 4 Department of Medical Biochemistry, St. Olav Hospital, Trondheim, Norway; 5 Multidisciplinary Unit of Biomedical Investigation (UMIB), Instituto de Ciências Biomédicas Abel Salazar (ICBAS), University of Porto, Porto, Portugal; 6 Internal Medicine, Pedro Hispano Hospital, Matosinhos, Portugal; 7 Evolutionary Systems Biology, Institute for Molecular and Cell Biology (IBMC), University of Porto, Porto, Portugal; 8 Molecular Evolution, Institute for Molecular and Cell Biology (IBMC), University of Porto, Porto, Portugal; 9 Department of Microbiology and Department of Epidemiology and International Health, University of Alabama at Birmingham, Birmingham, Alabama, United States of America; 10 Molecular Pathology and Immunology, Instituto de Ciências Biomédicas Abel Salazar (ICBAS), University of Porto, Porto, Portugal; Instituto de Ciencia de Materiales de Madrid - Instituto de Biomedicina de Valencia, Spain

## Abstract

Hereditary Hemochromatosis (HH) is a recessively inherited disorder of iron overload occurring commonly in subjects homozygous for the C282Y mutation in *HFE* gene localized on chromosome 6p21.3 in linkage disequilibrium with the human leukocyte antigen (HLA)-A locus. Although its genetic homogeneity, the phenotypic expression is variable suggesting the presence of modifying factors. One such genetic factor, a SNP microhaplotype named A-A-T, was recently found to be associated with a more severe phenotype and also with low CD8^+^T-lymphocyte numbers. The present study aimed to test whether the predictive value of the A-A-T microhaplotype remained in other population settings. In this study of 304 HH patients from 3 geographically distant populations (Porto, Portugal 65; Alabama, USA 57; Nord-Trøndelag, Norway 182), the extended haplotypes involving A-A-T were studied in 608 chromosomes and the CD8^+^ T-lymphocyte numbers were determined in all subjects. Patients from Porto had a more severe phenotype than those from other settings. Patients with A-A-T seemed on average to have greater iron stores (p = 0.021), but significant differences were not confirmed in the 3 separate populations. Low CD8^+^ T-lymphocytes were associated with HLA-A*03-A-A-T in Porto and Alabama patients but not in the greater series from Nord-Trøndelag. Although A-A-T may signal a more severe iron phenotype, this study was unable to prove such an association in all population settings, precluding its use as a universal predictive marker of iron overload in HH. Interestingly, the association between A-A-T and CD8^+^ T-lymphocytes, which was confirmed in Porto and Alabama patients, was not observed in Nord-Trøndelag patients, showing that common HLA haplotypes like A*01–B*08 or A*03–B*07 segregating with HFE/C282Y in the three populations may carry different messages. These findings further strengthen the relevance of HH as a good disease model to search for novel candidate loci associated with the genetic transmission of CD8^+^ T-lymphocyte numbers.

## Introduction

The major histocompatibility complex (MHC) region on chromosome 6p21.3 constitutes the most dense gene region of the human genome. It has been estimated that 40% of classical MHC genes are expressed in the immune system [1]. These genes are physically clustered, possibly reflecting functional relationships, and are characterized by high polymorphism levels and strong linkage disequilibrium. These characteristics make the MHC region a paradigm in many aspects of genomic research, particularly in disease association studies. Genetic variation in the MHC is associated with more disorders than any other genomic region, the majority of which are immune-related. Nevertheless, fine mapping of those disease associations and the identification of specific functional variants remain difficult. Both structural and regulatory variants are important in disease associations and may operate in tandem [1].

A classic example of disease association with extreme linkage disequilibrium at the MHC region is Hereditary Hemochromatosis (HH), an autosomal recessive disorder of primary iron overload characteristically found in Caucasians and associated with homozygosity for the *HFE* p.Cys282Tyr mutation (C282Y) in the vast majority of cases. *HFE* encodes a non-classical MHC class-I molecule and is localized 4 Mb telomeric to *HLA-A* [2], in very strong association with an ancestral haplotype carrying the human leukocyte antigen (HLA) antigens A*03 and B*07 [3], [4]. By applying several types of linkage-disequilibrium calculations to analyze the HH locus, Ajioka and co-workers found very high disequilibrium values over a large region from 150 kb centromeric to 5 Mb telomeric of *HLA-A*, partly due to an unusual low recombination rate of approximately 28% of the expected value [5]. In the same study, a haplotype phylogeny for HH chromosomes suggested that the origin of *HFE* C282Y is recent. These observations also provided a plausible explanation for previous difficulties in localizing the HH gene [6], [7].

Despite the genetic homogeneity at *HFE* among HH patients, their iron phenotypes are highly variable. Consequently, possible environmental and genetic modifiers of iron phenotypes in hemochromatosis have been intensively investigated. Among others, genes within the MHC class I region, inherited in linkage with the ancestral C282Y-containing haplotype, have been implicated in the clinical heterogeneity of *HFE*-associated HH [8]. However, conflicting results obtained by different authors have still not solved this question. Earlier independent studies in geographically different populations have shown that the number of copies of the common ancestral haplotype HLA-A*03–B*07 was associated with the expression of iron phenotypes. Patients with two copies of the ancestral haplotype were shown to have more severe iron overload phenotypes than those with one or no copy of the ancestral haplotype in studies performed in Australia, Italy and Alabama, USA [9]–[12]. Moreover, Pratiwi *et al*. showed by extended linkage disequilibrium analysis in patients from Australia that there are two distinct peaks of association separated by 2 Mb in the region of *HFE*, a pattern not expected for a single gene disorder [13]. This suggested that a gene modifying the phenotype of C282Y homozygotes could be localized around the area of D6S105. More recent studies did not support the previous observations [14]–[17]. In a review of the origin and spread of the hemochromatosis mutation, Distante and co-workers [14] reported that although associations of HLA haplotypes with the severity of iron overload were described, no such relationship was found in patients from the UK (R. Raha-Chowdhury, A. Bomford and M. Worwood, unpublished data). In another study of 8 HH families from Britanny, France, Sachot and co-workers analyzed C282Y homozygous relatives with no clinical signs of the iron overload in comparison to the respective probands who had abnormal iron phenotypes. They found no evidence that either *HFE* polymorphisms or variants in 10 microsatellite markers surrounding *HFE* could explain phenotypic variability in the respective kinships [15]. Barton and co-workers extended their study of genotype/phenotype correlations to a population of 141 C282Y homozygous probands from Alabama, USA, and did not reproduce the previous observations that were based on a relatively small number of probands [16]. Finally, in a study of HLA haplotypes of HH patients in a rural population from a former Norwegian province in Central Sweden, Olsson and co-workers could not find an association of the HLA-A*03 with the iron phenotype. Interestingly however, they found that males with double copies of the very common A*01–B*08 haplotype expressed a milder phenotype, supporting again an association of iron overload with the MHC region, but in the setting of a different haplotype [17].

Altogether the above described studies demonstrate that associations between the HH phenotype and the classical HLA markers vary among different cohorts from geographically distinct populations (who naturally diverge due to genetic drift or recombination events) and point to the necessity to look for novel markers at the MHC region that may help explaining the phenotypic variability in HH patients. One such factor could be a new 500 kb microhaplotype localized between *HFE* and the *HLA-A* locus as described by Cruz and coworkers [18]. This haplotype was associated with a more severe phenotype in its carriers and also with low CD8^+^ T lymphocyte numbers, which in previous studies from Portugal have predicted a more severe iron overload [19]–[22]. Low lymphocyte numbers were also associated with a more severe phenotype in patients from Alabama in particular those with HLA-A*01–B*08 [23]. However, this same haplotype reported in a former Norwegian province seemed to be associated with a milder phenotype [17]. Unfortunately, no data are available regarding CD8^+^T lymphocyte numbers in this population from Central Sweden.

In the present study we sought to test whether the predicting value of the microhaplotype described by Cruz *et al.* [18] could be reproduced in other settings, i.e., in different populations from geographically distant regions. In this context, we explored the degree of conservation of the reported HH-associated haplotypes in relation to their effect on the low CD8^+^ T lymphocyte phenotype or the clinical expression of iron overload. Our data indicate that although the same haplotypes are observed in distant geographical regions, their relative frequencies are variable, which may explain differences in genotype/phenotype associations among different populations.

## Methods

### Ethics Statement

The study was approved by the Ethical Committees of Centro Hospitalar do Porto, Porto; Institutional Review Board of Brookwood Medical Center, Alabama and The Regional Committee for Medical and Health Research Ethics, REC Central, Trondheim. Written informed consent was obtained from participants according to the Helsinki declaration.

### Study Populations & Clinical Data

Three different populations of Hereditary Hemochromatosis patients from geographically distant regions were included in this study. The only inclusion criteria for the purpose of the study were the confirmation of homozygosity for the C282Y *HFE* mutation and to be an adult, because the CD8^+^ T lymphocyte phenotype is stable in adults. The first group included 65 unselected, unrelated HH patients from the north of Portugal, mainly from the Porto district area, consecutively identified between 1985 and 2011 in non-screening settings and regularly followed up at the Hemochromatosis Outpatient Clinic of Santo António Hospital, Porto and Predictive and Preventive Genetic Centre, Porto. This group of patients is designated as Porto patients. The second group of patients included 57 unrelated HH patients diagnosed in non-screening settings from central Alabama, USA, diagnosed between 1988 and 2010 and treated at Southern Iron Disorders Center, Birmingham, Alabama. These probands were selected for the present study only because they presented for diagnosis or treatment in a consecutive mode. This group of patients is designated as Alabama patients. The third group of patients included 182 patients from the Nord-Trøndelag County, Norway, who were diagnosed with HH as part of a population screening study (HUNT2) between 1995 and 1997, and were followed up at St. Olav Hospital, Trondheim. This group of patients is designated as Nord-Trøndelag patients. Most of the clinical and laboratory information about all patients were already available and described elsewhere [8], [18], [24]–[28]. Previously available information included, in all patients, the iron parameters at diagnosis: transferrin saturation (TfSat) and serum ferritin (SF); *HFE* genotype (all homozygous for the C282Y mutation); and *HLA* class I alleles (A and B) as determined by low-resolution DNA-based techniques (PCR/sequence-specific oligonucleotide probes, Dynal RELI™ SSO, Dynal Biotech Ltd, UK). Values of total body iron stores (TBIS) estimated by quantitative phlebotomies were available from 104 patients (34 from Porto, 32 from Alabama and 38 from Nord-Trøndelag).

### Immunophenotyping

Blood counts of T-CD8^+^ lymphocyte subpopulation were available for all study participants. T-lymphocyte subpopulations were determined by FACS analysis using anti-CD3 and anti-CD8 monoclonal antibodies as previously described in detail [24]. We defined as a “low CD8 phenotype” the finding of CD8^+^ T lymphocyte numbers below the 25% percentile in controls. This value was 310×10^3^/ml in Porto and Alabama patients and 319×10^3^/ml for Nord-Trøndelag patients. Mean values (± standard deviation) in controls were 433(±168) x10^3^/ml in Porto and Alabama and 490(±234) x10^3^/ml in Nord-Trøndelag.

### Genetic Markers at the MHC Region

In addition to *HFE* and *HLA* genotyping, genetic information on three single nucleotide polymorphisms (SNPs) localized in the region between *HFE* and *HLA-A* was obtained in all patients included in this study. These SNPs were localized in the genes: piggyBac transposable element derived 1 (*PGBD1, rs1997660*), zinc finger protein 193 (*ZNF193, rs7206*) and zinc finger protein 165 (*ZNF165, rs203878*), and defined a SNP microhaplotype of 500 kilobases (kb). These were the SNP microhaplotypes previously described in Porto patients [18] and were determined “de novo” in patients from Alabama by gene sequencing as described in [18] and in patients from Nord-Trøndelag by hybridization probe melting curve analysis on the LightCycler®, Roche Diagnostics. Details on primer and probe sequences in addition to PCR conditions for SNP analysis can be provided by request.

### Generation of Phased Chromosomes and Haplotype Construction

*HLA A–B* haplotypes, and the SNP microhaplotypes defined by the genes *PGBD1, ZNF193* and *ZNF165,* were defined in HH patients by family segregation whenever informative family members were available. Otherwise they were inferred by the PHASE program.

### Statistical Methods

Associations of *HLA* alleles and haplotypes in chromosomes carrying the C282Y mutation in *HFE* were tested by the Chi-square test by comparison of their frequencies in HH patients from the 3 different regions of Porto, Alabama and Nord-Trøndelag with those of the respective reference normal populations. For the purpose of statistical analysis, only alleles or haplotypes with frequencies respectively higher than 10% and 7% in any of the tested population were considered. Information about HLA allele and haplotype reference frequencies in the normal populations from Porto (north Portugal) was obtained at the “Allele*Frequencies in worldwide populations” database [30]. Frequencies for the Alabama control population were reviewed from the data previously analyzed by Barton and co-workers [26]. Frequencies in the non-Sami population from Norway were obtained from the data described by Harbo *et al.* 2009 [29], also published at the “Allele*Frequencies in worldwide populations” database [30]. In order to eliminate the artificially lowered frequencies in HH chromosomes of other alleles and haplotypes that were due to the relatively high frequencies of alleles A*03 and B*07 and the haplotype A*03–B*07, we estimated (in patients and respective controls) “corrected” allele and haplotype frequencies by subtracting from the denominator respectively the sum of A*03 and B*07 alleles or the number of A*03–B*07 haplotypes. These corrected frequencies allowed a more meaningful comparison between frequencies in HH and control chromosomes, as originally described by Marcel Simon and co-workers [4]. To analyze the relative strength of HLA allele or haplotype associations, the etiological fraction delta (δ) was calculated as described according to the formula δ = (FAD-FAP)/(1-FAP) where FAD is the allele frequency in HH chromosomes and FAP the allele frequency in control chromosomes [31]–[33]. In the case of multiple comparisons we used the Bonferroni correction to test for the significance of differences.

To investigate the association of CD8^+^ T lymphocyte numbers with particular genotypes, we assigned to each chromosome the value of CD8^+^ T lymphocytes of the respective carrier. Differences in mean CD8^+^ T lymphocyte values among groups were tested by the Student’s T-test or the One-Way analysis of variance (ANOVA) as appropriate. In addition, patients with CD8^+^ T lymphocyte numbers below the 25% percentile of the respective controls were selected and their chromosomes assigned as “low CD8 phenotype” cases. Differences in the relative frequencies of “low CD8 phenotype” cases among groups were tested by the Chi-square test.

Quantitative measures of iron parameters were also compared among the 3 populations of HH patients from Porto, Alabama and Nord-Trøndelag. Because of skewness in the distribution of serum ferritin and total body iron stores, for statistical purposes the logarithmic transformation was applied to those values. For representation in table and figure, however, the non-transformed values were used. Differences in means among groups were tested by One-Way analysis of variance (ANOVA) or the Student’s T-test as appropriate.

Data were analyzed by Statgraphics software (Statgraphics Graphics System, version 7.0). Values of *P*<0.05 were defined as significant.

## Results

### 1. Clinical Heterogeneity among the HH Populations from Porto, Alabama and Nord-Trøndelag

A summary of the iron-related parameters of the HH patients from Porto, Alabama and Nord-Trøndelag is provided in Table 1, where values are given according to gender. Significant differences among the 3 populations of patients were observed in males for TfSat, SF, and TBIS, with *P* values <0.00001 in all cases. These differences were explained by a more severe expression in patients from Porto and a milder expression in Nord-Trøndelag patients. In females, SF (*P<*0.00001) and TBIS (*P<*0.04) were also significantly different among the 3 populations, with Porto and Nord-Trøndelag patients having respectively the highest and lowest values. The HH cohort from Nord-Trøndelag was the only one in which patients were identified in screening programs. Moreover, previous studies in the same population showed that, in general, Nord-Trøndelag patients have a low prevalence of clinical symptoms and less severe iron overload [27], [28].

**Table 1:** Iron parameters (at diagnosis) in HH patients from Porto, Alabama and Nord-Trøndelag.

	N	TfSat (%)	SF (ng/ml)	TBIS (g)
**HH male patients from:**				
** Porto**	43	90±14 (63–123)	1750±295 (163–7685)	7.93±0.78 (2.19–17.40)
** Alabama**	32	73±17 (41–100)	815±82 (123–2119)	3.66±0.50 (0.40–10.40)
** Nord-Trøndelag**	103	81±9 (58–100)	541±63 (27–3511)	3.23±0.47 (1.12–15.32)
* P value*		*<0.00001*	*<0.00001*	*<0.00001*
**HH female patients from:**				
** Porto**	22	81±18 (55–111)	543±286 (67–3954)	3.20±1.33 (1.10–13.80)
** Alabama**	25	74±20 28–100)	433±78 (65–1892)	1.93±0.27 (0.40–5.60)
** Nord-Trøndelag**	79	73±12 (51–97)	172±27 (16–1151)	1.65±0.30 (0.89–4.32)
* P value*		*n.s.*	*<0.00001*	*0.040*

Transferrin saturation (TfSat) is presented as arithmetic mean ± standard deviation; serum ferritin (SF) and total body iron stores (TBIS) are presented as geometric mean ± standard error. Minimum-maximum values are in parenthesis. TBIS was available in 34 males from Porto, 32 from Alabama and 38 from Nord-Trøndelag and in 13 females from Porto, 23 from Alabama and 12 from Nord-Trøndelag.

Statistically significant differences (*P value indicated*) were tested among groups using One-way Anova.

### 2. Analysis of Genetic Markers between *HFE* and *HLA-B* in the HH Populations from Porto, Alabama and Nord-Trøndelag

From the study of 304 HH patients with C282Y homozygosity from three geographically distant regions, namely Porto, Portugal (n = 65), Alabama, USA (n = 57) and Nord-Trøndelag, Norway (n = 182), we obtained respectively 130, 114 and 364 chromosomes carrying *HFE* C282Y. These were genetically characterized with 5 different markers including *HLA-A* alleles, *HLA-B* alleles, and SNPs in the genes *PGBD1, ZNF193* and *ZNF165*. HLA A–B haplotypes and SNP microhaplotypes were assigned by family segregation, or generated by PHASE in patients without available informative family members (see Methods).

HLA-A and HLA-B allele frequencies were first analyzed in HH chromosomes from the three populations. Results are summarized in Table 2 (uncorrected data are shown in Table S1). As expected from all previously published studies of HLA associations in HH, the most common HLA-A and B alleles in chromosomes from all the three HH populations from Porto, Alabama and Nord-Trøndelag were A*03 (respectively 0.408, 0.474 and 0.420) and B*07 (respectively 0.238, 0.307 and 0.288); these frequencies were significantly different from those of the corresponding controls (Table 2). The strength of these significant associations was measured by estimating the etiological fraction delta (see Methods) being similar in all populations (Table 2). After correcting for the strong effect of A*03 and B*07 on other allele frequencies (see Methods), other significantly associated HLA alleles were found in the populations from Porto (A*01, B*08 and B*40) and Nord-Trøndelag (A*11, B*14 and B*44). In Alabama patients, the only additional HLA allele with a statistically significant association was B*14, suggesting that Alabama patients represent a genetically more conserved population.

**Table 2:** HLA allele and haplotype associations in *HFE* C282Y carrying chromosomes of HH patients from three populations.

	Porto				Alabama				Nord-Trøndelag			
	HH Patients (n = 130 chromosomes)	Controls*(n = 15874 chromosomes)			HH Patients (n = 114 chromosomes)	Controls*(n = 830 chromosomes)			HH Patients (n = 364 chromosomes)	Controls *(n = 1152 chromosomes)		
HLA-	*allele frequency*		*P value*	δ	*allele frequency*		*P value*	δ	*allele frequency*		*P value*	δ
**A** * **03**	**0.408**	**0.101**	**0.000**	**0.330**	**0.474**	**0.166**	**0.000**	**0.369**	**0.420**	**0.155**	**0.000**	**0.310**
**B** * **07**	**0.238**	**0.060**	**0.000**	**0.180**	**0.307**	**0.130**	**0.000**	**0.203**	**0.288**	**0.148**	**0.000**	**0.160**
	*corrected allele frequency* **				*corrected allele frequency* **				*corrected allele frequency* **			
**A** * **01**	**0.208**	**0.124**	**0.026**	**0.096**	0.283	0.214	**ns**		0.223	0.180	ns	
**A** * **02**	0.299	0.295	ns		0.300	0.328	ns		0.360	0.406	ns	
**A** * **11**	0.052	0.078	ns		0.067	0.068	ns		**0.137**	**0.052**	**0.000**	**0.090**
**A** * **24**	0.065	0.117	ns		0.067	0.082	ns		0.100	0.061	ns	
**B** * **08**	**0.131**	**0.079**	**0.053**	**0.057**	0.165	0.152	ns		0.127	0.143	ns	
**B** * **14**	0.051	0.072	ns		**0.139**	**0.040**	**0.0001**	**0.103**	**0.100**	**0.032**	**0.000**	**0.071**
**B** * **15**	0.020	0.061	ns		0.025	0.005	ns		0.085	0.130	ns	
**B** * **35**	0.162	0.126	ns		0.051	0.094	ns		0.077	0.107	ns	
**B** * **40**	**0.101**	**0.035**	**0.000**	**0.068**	0.013	0.014	ns		0.135	0.132	ns	
**B** * **44**	0.162	0.162	ns		0.203	0.163	ns		**0.228**	**0.159**	**0.000**	**0.082**
	*haplotype frequency*				*haplotype frequency*				*haplotype frequency*			
**A** * **03B** * **07**	**0.169**	**0.013**	**0.000**	**0.158**	**0.272**	**0.033**	**0.000**	**0.247**	**0.214**	**0.060**	**0.000**	**0.164**
	*corrected haplotype frequency* **				*corrected haplotype frequency* **				*corrected haplotype frequency* **			
**A** * **03B** * **14**	0.019	n.a.			**0.096**	**0.009**	**0.000**	**0.089**	0.077	n.a.	n.a	
**A** * **01B** * **08**	0.065	0.034	ns		0.108	0.069	ns		0.098	0.096	ns	
**A** * **02B** * **44**	0.037	0.038	ns		0.096	0.062	ns		0.080	0.075	ns	

Comparisons between patients and controls were done using the Chi-square test (*P* values indicated) and the strength of the associations was estimated by the etiological fraction delta (**δ**).

* HLA allele and haplotype frequencies in the controls populations from Porto (north Portugal) were obtained at the “Allele Frequencies in worldwide populations” database (Gonzalez-Galarza *et al*.(2011) Nucleic Acids Res 39∶913–919), from Alabama were reviewed from previous data reported by Barton et al. (2002) BMC Med Genet 3∶9; and from Norway were obtained in the study by Harbo *et al.* (2009) Tissue Antigens 75∶207–217.

** Corrected allele and haplotype frequencies (see Methods) were calculated by subtracting from the denominator, respectively, the sum of A*03 and B*07 alleles and the number of A*03B*07 haplotypes.

n.a. = data not available; ns = not significant.

The most prevalent HLA A–B haplotype in the three populations from Porto, Alabama and Nord-Trøndelag was A*03–B*07, the proportion of its carriers being 0.169, 0.272 and 0.214, respectively (see also Table 2). Although these haplotype frequencies do not differ statistically among the different populations (shown in Supplementary Table 1), the strength of their associations to HH, as measured by the etiological fraction delta, is stronger in Alabama (δ = 0.247) than in Nord-Trøndelag (δ = 0.164) or Porto (δ = 0.158), suggesting a more recent founder effect in Alabama HH patients. This interpretation is also consistent with our observation that the prevalence of HLA-A and -B alleles and haplotypes is less diverse in Alabama patients than in Porto or Nord-Trøndelag patients. After we corrected for the predominance of A*03–B*07 haplotypes on other haplotype frequencies (see Methods), the next most common HLA haplotype found in patients from all populations was A*01–B*08, although the respective frequencies of A*01–B*08 did not differ significantly from those in the respective control populations. The A*03–B*14 haplotype also occurred in hemochromatosis chromosomes from Alabama and Nord-Trøndelag patients. The significance of this association could be defined only in the Alabama population due to lack of sufficient available information about control subjects in Porto and Nord-Trøndelag. Nevertheless, its significance might be supported by the very low frequencies found in controls from Scandinavia [34]. The haplotype A*02–B*44, a very common haplotype in normal Caucasian populations, was found at similar frequencies in all non-A*03–B*07-carrying chromosomes from the three present populations. These respective frequencies did not differ significantly from those in the respective control subjects.

We next analyzed the SNP microhaplotypes defined by the SNPs in the genes *PGBD1, ZNF193* and *ZNF165* in the three populations (results shown in Table S1). Previous studies in HH patients had revealed that the most conserved of these SNP microhaplotypes was the one designated as A-A-T and that this microhaplotype is also transmitted in association with a more severe iron phenotype of HH [18]. This microhaplotype was the most prevalent in all the populations studied here but its relative frequency differed significantly among them (*P* = 0.0003). Porto patients had the highest A-A-T frequency (0.908) and Nord-Trøndelag patients the lowest (0.765). Among the non-A-A-T SNP microhaplotypes, the most common was G-G-G, the frequency of which also differed significantly among the three populations (*P* = 0.003). G-G-G frequency was highest in Nord-Trøndelag patients (0.160) and lowest in Porto patients (0.062).

In summary, the observation of differences in the relative frequencies of the described HLA and SNP markers among the three geographically distant populations of HH patients support the postulate that evolutionary histories among populations differ due to differences in genetic drift and recombination of the HH founder chromosomes in the respective geographic regions.

### 3. Haplotype Conservation in HH Patients from Porto, Alabama and Nord-Trøndelag

**Figure 1:**
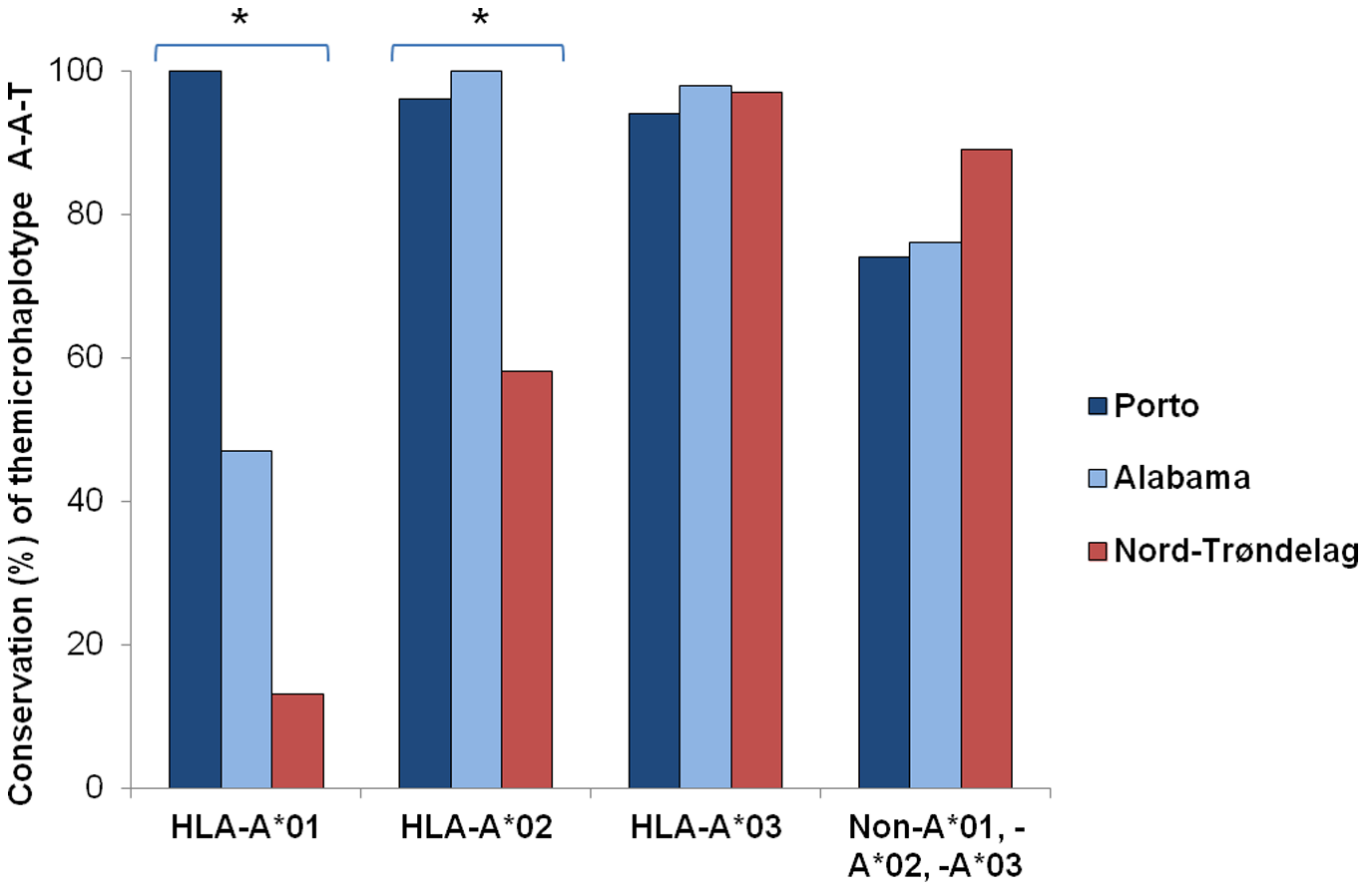
Conservation (%) of the SNP microhaplotype A-A-T according to HLA-A alleles in chromosomes from HH patients. A comparison of the percent haplotype conservation among the three groups of HH patients from Porto, Alabama and Nord-Trøndelag was done using the Chi-square test and significant results are indicated by a * (*P*<0.00001).

We analyzed the degree of haplotype conservation as a measure of their proximity from the ancestral HH founder chromosomes by selecting the HH chromosomes carrying the most commonly associated HLA-A alleles (A*01, A*02 and A*03) and calculating the degree of conservation (%) of the most common SNP microhaplotype A-A-T in those chromosomes. Results are shown in Table 3 and Figure 1. In general, a high degree of conservation was observed in cohorts from Porto, Alabama and Nord-Trøndelag for chromosomes carrying A*03 (respectively 94%, 98% and 97%), including the A*03–B*07-carrying chromosomes (respectively 91%, 100% and 99%). In contrast, significant differences occurred among the three populations regarding the conservation of chromosomes carrying A*01 or A*02 alleles (Table 3). These differences are illustrated in Figure 1, particularly visible for chromosomes carrying HLA-A*01. In the case of Porto patients, the SNP microhaplotype A-A-T is conserved in all HLA-A*01 carrying chromosomes (16/16), including all HLA-A*01–B*08 carrying chromosomes (7/7). This was not observed in either Alabama or Nord-Trøndelag. In the case of Alabama patients, 53% (9/17) of HLA-A*01-carrying chromosomes (or 56%, 5/9, of A*01–B*08 carrying chromosomes) do not conserve the A-A-T microhaplotype. In Nord-Trøndelag patients, 87% (41/47) of chromosomes carrying HLA-A*01 (or 86%, 25/29, of A*01–B*08 carrying chromosomes) do not conserve the A-A-T microhaplotype. In the Nord-Trøndelag population the A-A-T microhaplotype was not conserved in 42% (32/76) of A*02 carrying chromosomes. In contrast, the percentage of conservation was 96% (22/23) and 100% (18/18) in Porto and Alabama cohorts, respectively. Taken together, the present results further suggest that the recombination histories or founder effects of the C282Y-carrying HH chromosomes differ in the three hemochromatosis populations studied. This may affect other traits encoded in the same chromosomal region, including determinants of CD8^+^ T lymphocyte numbers or other putative modifiers of iron overload.

**Table 3:** Comparison of the conservation of the SNP microhaplotype A-A-T in chromosomes of HH patients from Porto, Alabama and Nord-Trøndelag.

		Percentage (n) of haplotypes			
Associated HLA alleles	Associated SNP microhaplotype	Porto	Alabama	Nord-Trøndelag	*P* *
**A** * **01**					
	**Conserved A-A-T**	100% (16)	47% (8)	13% (6)	2.52×10^−9^
	**Non conserved A-A-T**	0	53% (9)	87% (41)	
**A** * **02**					
	**Conserved A-A-T**	96% (22)	100% (18)	58% (44)	3.06×10^−5^
	**Non conserved A-A-T**	4% (1)	0	42% (32)	
**A** * **03**					
	**Conserved A-A-T**	94% (50)	98% (53)	97% (148)	n.s.
	**Non conserved A-A-T**	6% (3)	2% (1)	3% (5)	
**Non A** * **01–A** * **02–A** * **03**					
	**Conserved A-A-T**	74% (28)	76% (19)	89% (78)	n.s.
	**Non conserved A-A-T**	26% (10)	24% (6)	11% (10)	

* Relative frequencies of conserved and non-conserved haplotypes among three populations were compared using the Chi-square test (*P values* are indicated).

### 4. Associations of the CD8^+^ T Lymphocyte Phenotype with MHC Markers in HH Patients

**Figure 2:**
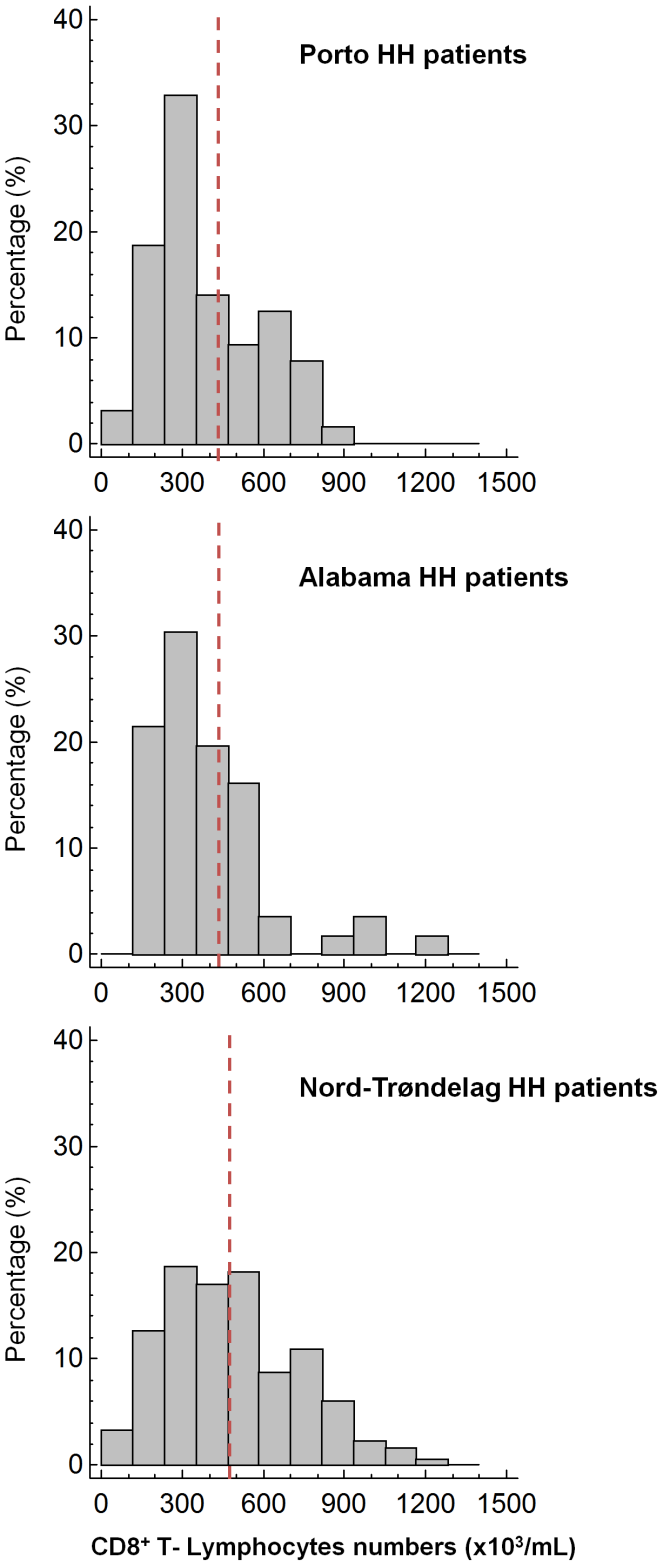
Distribution of peripheral blood CD8^+^ T lymphocytes in HH patients from Porto, Alabama and Nord-Trøndelag. The dash lines indicate the mean value observed in the respective control populations.

We sought to investigate the association of CD8^+^ T lymphocyte numbers with particular MHC markers. First, we analyzed the distribution of CD8^+^ T lymphocyte numbers in the three populations of HH patients, each of whom had C282Y homozygosity. Low CD8^+^ T lymphocyte numbers were common in patients from each geographic region (Fig. 2), but the distribution of T lymphocyte numbers differed. Patients from Porto and Alabama had a more striking deviation to low numbers than patients from Nord-Trøndelag (Fig. 2).

We then analyzed the associations of the “low CD8 phenotype” with particular extended haplotype combinations among the three different populations. A “low CD8 phenotype” was defined as CD8^+^ T lymphocyte numbers below the 25% percentile in the respective controls, i.e., 310×10^3^/ml for Porto and Alabama, and 319×10^3^/ml for Nord-Trøndelag (see Methods). The extended haplotype combinations were chosen to reflect the degree of conservation relative to the most common ancestral haplotype, i.e., if the A-A-T microhaplotype was conserved or not and, within A-A-T conserved haplotypes, if HLA*-*A*03 was conserved or not (Table 4). We estimated the frequencies of “low CD8 phenotype” cases for each haplotype combination. The results, illustrated in Table 4, demonstrate that there are differences among the three populations. The “low CD8 phenotype” was significantly associated with the most conserved haplotypes carrying A-A-T and the HLA-A*03 in the populations from Porto (*P = *0.045) and Alabama (*P* = 0.012) but not in the population from Nord-Trøndelag. These associations were also reflected on the mean CD8^+^ T lymphocyte counts. Values in Porto and Alabama patients were significantly lower than the expected values in controls (*P = *0.0017 and *P = *0.021, respectively). Taken together, the different patterns of association of the “low CD8 phenotype” with particular extended haplotype combinations in the three populations of patients suggest a stronger founder effect in the patients from Porto and Alabama with fewer recombination events between a putative locus marking the “low CD8 phenotype.” In patients from Nord-Trøndelag, genotype/phenotype associations were apparently lost.

**Table 4:** Correlations between haplotype conservation and the CD8^+^ T lymphocyte phenotype in chromosomes of HH patients from Porto (n = 128), Alabama (n = 112) and Nord-Trøndelag (n = 362).

	Relative frequency of the “low CD8 phenotype”				Mean (±SD) of CD8+ T lymphocytes(x10^3^/ml)			
Extended haplotype combinations	Porto HH patients	Alabama HH patients	Nord-Trøndelag HH patients	P value	Porto HH patients	Alabama HH patients	Nord-Trøndelag HH patients	P value
Conserved-A-A-T With HLA-A*03	44.0% (22/50)*	49.0% (25/51)*	25.7% (38/148)	0.0026	370±179*	369±233*	507±251	0.0001
Conserved-A-A-T Without HLA-A*03	39.4% (26/66)	31.1% (14/45)	38.3% (49/128)**	n.s.	393±194	453±263	458±259	n.s.
**Non- conserved-A-A-T**	16.7% (2/12)	43.8% (7/16)	31.4% (27/86)	n.s.	540±207	430±331	469±216	n.s.

The percentage (case/total numbers) of patients with CD8^+^ T lymphocytes below the 25% percentile (“low CD8 phenotype”) are indicated followed by the mean (±standard deviation) of CD8^+^ T lymphocyte counts (x10^3^/ml) for each haplotype combination.

(*P*) Statistical significant differences among the 3 populations of patients (using the Chi-square test or One-way ANOVA, as appropriate, see M&M).

(*) Results significantly lower (p<0.05) than the respective control populations (using the Chi-square test or Student T-test, as appropriate, see M&M).

(**) Result significantly lower than the respective control due to a small (n = 5) founder group of HLA-A*01 patients. The statistical significance is lost if this group is excluded.

### 5. Associations of the Iron Phenotype with MHC Markers in HH Patients

**Figure 3:**
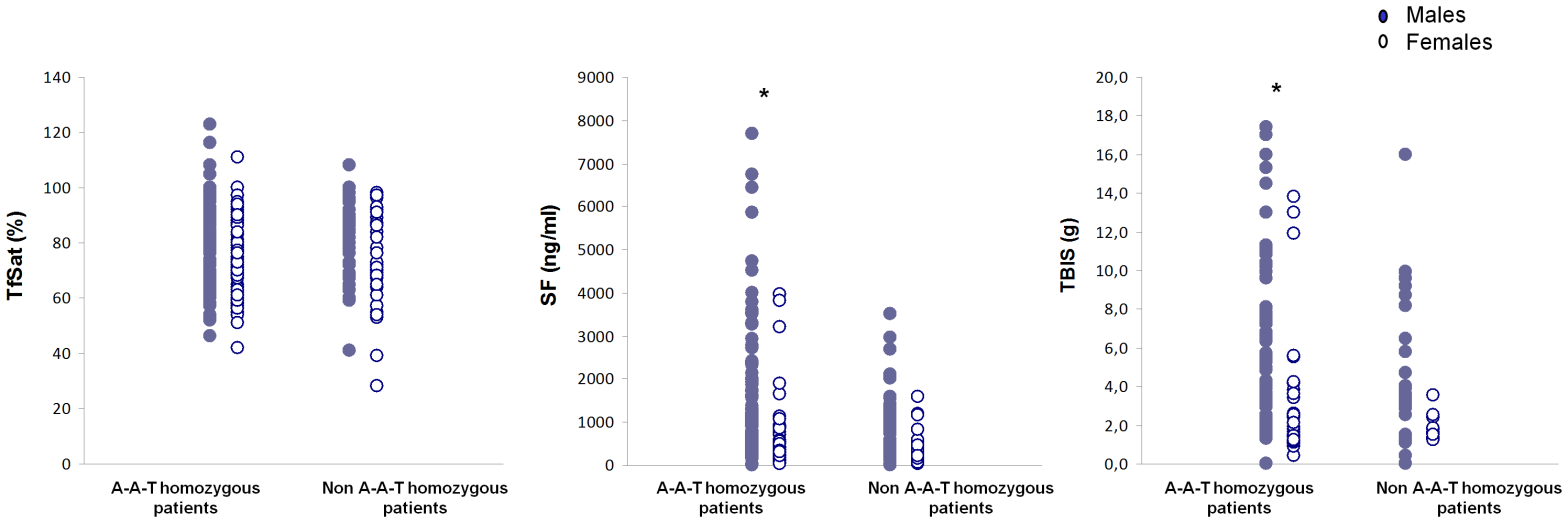
Effect of the SNP microhaplotypes on the expression of iron overload. Comparisons of the iron parameters: transferrin saturation (TfSat), serum ferritin (SF) and total body iron stores (TBIS) between groups of HH patients divided according to the associated SNP microhaplotypes (A-A-T homozygous or non-A-A-T homozygous). Males are represented by solid circles and females represented by open circles. Significant differences in the mean values (by the Student´s T test) are indicated by an *(*P = *<0.027).

In order to analyze the effect of associated SNP microhaplotypes on the clinical expression of iron overload, HH patients were divided in two groups, according to the presence, in homozygosity, of the ancestral SNP microhaplotype A-A-T. For statistical purposes, males and females were analyzed separately. Results are presented in Table 5 and Figure 3. No significant differences were found in females and no significant differences were found for TfSat in both males and females (Fig.3). In general, the average SF and TBIS values in male patients were significantly higher (respectively *P = *0.027 and *P = *0.021) in those homozygous for the A-A-T microhaplotype than in those carrying one or more non-A-A-T microhaplotype. Even if the average SF and TBIS values in male patients appeared higher in those homozygous for the A-A-T, no significant differences were seen in the 3 separate populations (Table 5). In conclusion, these results may support a general prediction of a more severe iron phenotypes in patients’ populations carrying the conserved A-A-T microhaplotype in homozygosity, but they also show that, for individual purposes, the microhaplotype A-A-T cannot be used as a universal marker of iron phenotype in HH.

**Table 5:** Average values of total body iron stores (TBIS) and serum ferritin (SF) of HH male patients according to the associated SNP microhaplotypes (A-A-T homozygous or non- A-A-T homozygous).

	All HH patients	HH patients from		
		Porto	Alabama	Nord-Trøndelag
**Average of TBIS (g) in:**				
A-A-T homozygous male patients	**4.98 [4.24–5.85] (n = 77)**	8.08 [6.42–10.11] (n = 29)	3.83 [3.01–4.88] (n = 24)	3.62 [2.75–4.76] (n = 24)
Non-A-A-T homozygous male patients	**3.37 [2.43–4.69] (n = 27)**	7.11 [3.07–16.48] (n = 5)	3.23 [1.34–7.75] (n = 8)	2.65[1.87–3.77] (n = 14)
*P* *	***0.021***	*n.s.*	*n.s.*	*n.s.*
**Average of SF (ng/ml) in:**				
A-A-T homozygous male patients	**849 [718–1004] (n = 124)**	1764 [1296–2400] (n = 36)	818 [670–999] (n = 24)	571 [461–707] (n = 64)
Non-A-A-T homozygous male patients	**602 [470–771] (n = 51)**	1652 [576–4737] (n = 5)	806 [402–1616] (n = 8)	496 [381–646] (38)
*P* *	***0.027***	*n.s.*	*n.s.*	*n.s.*

TBIS and SF are presented as geometric mean and 95% Confidence Interval for Mean [Lower Bound - Upper Bound]; the numbers of patients in each group (n) are indicated in each case.

* (*P*) Statistical significant differences using the Student´s T- test (with log transformed values, see methods).

## Discussion

The question of HLA haplotype conservation in HH has been a focus of scientific interest for a long time, and several interpretations about its role in the recent evolutionary history of chromosomes carrying the C282Y mutation have been largely discussed [3]–[7], [14], [17], [35], [36]. Besides the well described A*03–B*07 ancestral haplotype [3]–[7], also the A*01–B*08 haplotype is very long and resistant against recombination, and appears to be derived from a single ancestor [14], [17]. The present study explored the implications of haplotype conservation on HH patient’s phenotypes. While not confirming the value of the A-A-T microhaplotype as a universal predictive marker of iron overload in HH, the study revealed important differences in both the genetic composition and the genotype/phenotype correlations among the geographically distant populations which may help explaining differences in phenotype and local penetrance of the disease. The most relevant questions raised by these results were: Why do all A*01–B*08 haplotypes from Porto patients carry the conserved A-A-T microhaplotype, but only 47% in Alabama and 13% in Nord-Trøndelag? Why don’t we see an association of the A-A-T microhaplotype with low CD8^+^ T lymphocytes in the Nord-Trøndelag population, as we observe in Porto and Alabama? Could the selection of “non-conserved” chromosomes in *HFE* C282Y homozygotes may help us in future to identify the individual loci contributing to the “low CD8 phenotype” and/or other novel associated modifiers of iron overload? Could the loss of A-A-T microhaplotype provide the explanation for the mild phenotype of A*01–B*08 carriers in particular populations, such as the one described in a former Norwegian province in Sweden [17]?.

In a recent study, Baschal and co-workers analyzed HLA data and genotypes for thousands of SNPs across the MHC complex in a large number of families, demonstrating the occurrence of multiple common “completely” conserved complex SNP haplotypes in the MHC region, several of them influencing disease susceptibility [37]. They suggested that such conservation could also occur in other genomic areas and proposed that this type of analysis of conservation versus sub-conservation of extended haplotypes may be an important tool for further positioning of disease-associated loci. In the present study, we took advantage of the known occurrence of highly conserved MHC-linked haplotypes in patients with HH and its known association with a phenotype of low CD8^+^ T lymphocyte numbers to study the distribution and composition of the HH-associated chromosomes and explain differences found in genotype/phenotype correlations among three geographically distant populations. Although high frequencies of the low CD8^+^ T lymphocyte phenotype were found in all HH populations, the pattern of association of this phenotype with particular haplotypes differed among patients from the three geographic regions, possibly reflecting diverse haplotype structures due to different recombination histories or founder effects.

### Haplotype Heterogeneity among Populations

The simple analysis of the distribution of *HLA* associated haplotypes (Table 2), in addition to the degree of conservation of the associated A-A-T SNP microhaplotype (Table 3, Fig. 1) in the three cohorts of patients from Porto, Alabama and Nord-Trøndelag, shows that the strong association of HH with the HLA-A*03–B*07 haplotype is the most consistent observation in all populations studied, confirming the existence of a common ancestral haplotype subsequently modified by recombination and geographical scattering due to migrations [4]. The diversity of associations with other haplotypes reveals differences among populations which agree with the expected differences in the history of their HH-carrying chromosomes, taking into consideration particular founder effects or the time for recombination events. The most stricking differences are observed in chromosomes carrying HLA-A*01 (which include the ancestral HLA-A*01–B*08), in which the SNP microhaplotype A-A-T was conserved in 100% chromosomes of Porto patients, while it was less conserved in patients from Alabama (47%) and much less conserved in or Nord-Trøndelag patients (13%) (see Fig.1), supporting the different founder effects or distinct recombination histories in the respective populations. The highest haplotype diversity observed in the Nord-Trøndelag population is also consistent with the high frequency of *HFE* C282Y in Norwegians [27], [28], [38], possibly related with characteristics of rapid population growth that has occurred in northwestern Europe since the Celtic period [14]. On the contrary, patients from Alabama showed the lowest haplotype diversity, reflecting a more recent founder effect. A previous study showed that aggregate “British Isles” or Scotland indices of ancestry were significantly greater and the proportion of non-British Isles, non-Native American ancestry was significantly lower in Alabama hemochromatosis probands with *HFE* C282Y homozygosity than in population control subjects [39], [40]. These observations suggest that British Isles ancestry likely accounts for the relatively high C282Y allele frequency and association of HLA-A*03–B*07 and *HFE* C282Y in central Alabama whites. Therefore, the evidence of a recent founder effect in Alabama HH patients suggested by the present results agrees with the previous ancestry studies and with the predominance of English people among whites who migrated to and settled the geographic area of the present State of Alabama in the late 18th and early 19th centuries [41], [42]. HLA-A*03–B*14 also occurred in hemochromatosis chromosomes from Alabama and Nord-Trøndelag patients. Although this haplotype is also described as a common HH ancestral haplotype in hemochromatosis populations in many northwestern European countries, particularly in Scandinavia [36], its appearance in Alabama HH patients is unlikely to be attributed predominantly to Norwegian or other Scandinavian founders because ancestry reports from these geographic areas of Europe are rare in Alabama hemochromatosis probands and population control subjects [39]. Nevertheless this haplotype could have a common ancestral Irish origin and be spread in Norway by the close contacts between Ireland and Scandinavia through the Vickings’ movements [35], [36]. In the case of Porto patients, the relative low diversity of HH haplotypes could be attributed to the particular demographic characteristics of the Portuguese population in the north region namely the unipolar mode of migration and the low rate of mobility from other regions [43]. Significant regional differences were previously found in the distribution of the C282Y mutation in Portugal with the highest frequencies found in the north of the country [44]. In terms of historical population settlements, it is well recognized that there is a geographical and cultural boundary between the north and the south of Portugal documented by archeological, ethnographic and linguistic records [43], all favoring the notion that a stronger Celtic influence in the north could map the founder HH chromosomes in this region by the 6^th^ century BC. The hypothesis that the later Nordic/Suevian occupation and settlement, which also occurred only in the north of the present country, could also contribute to the increased frequency of the mutation cannot be excluded. The present results of divergent patterns of haplotype conservation and genotype/phenotype associations in patients from Porto and Nord-Trøndelag do not favor a strong Scandinavian HH founder effect in north Portugal.

### Effect of Haplotype Conservation on the CD8^+^ T Lymphocyte Phenotype

It is well known that the existence of different founder effects and different recombination histories at the MHC region affect the transmission of other genetic traits encoded in the same chromosomal region [1]. The previously demonstration that CD8^+^ T lymphocyte numbers are transmitted in association with particular HLA haplotypes in Portuguese HH patients [24], [25] prompt us to analyze if the same association was also observed in the other HH populations, We confirmed that the phenotype of low CD8^+^ T lymphocytes was commonly observed in each of the three populations, but their respective distributions (Fig. 2) and their genotype/phenotype correlations (Table 4) varied. The “low CD8 phenotype” was significantly associated with the most conserved ancestral haplotype carrying A*03-A-A-T in the cohorts from Porto (n = 50) and Alabama (n = 51) but it was not associated in a much greater series from Nord-Trøndelag (n = 148). This lack of association of the common ancestral haplotype with the “low CD8 phenotype” in Nord-Trøndelag patients is intriguing. One should stress however, that discrepancies in expected genotype/phenotype correlations can also be highly informative regarding the localization of genetic traits. That individual chromosomes with the same alleles (A*03, A-A-T) may or may not be associated with the “low CD8 phenotype” indicates that these alleles are not, by themselves, determinants of the trait, further supporting the hypothesis of another independent, still unidentified, genetic marker in the region. The time and place, during the evolutionary history of the HH chromosomes, when the association occurred remains unknown. Further studies of genotype/phenotype correlations in other populations with different founder effects could clarify this question. As suggested by Olsson and co-workers [35], it would be of great interest to explore further the HLA haplotype/phenotype correlations in an extended population of patients from the Trøndelag region, because of its long historic close contacts with the British islands, the supposed origin of founders of the “Celtic” haplotypes in Scandinavia [35]. On the other hand, results from Porto and Alabama support the postulate that a major genetic determinant of CD8^+^ T lymphocyte numbers is transmitted in linkage disequilibrium with *HFE* in this ancestral haplotype and suggest these populations as good targets to further position a candidate locus associated with the transmission of low CD8^+^ T lymphocyte phenotypes. The evidence that the association is lost not only in chromosomes without the A-A-T SNP microhaplotype but also, within the A-A-T conserved haplotypes, in chromosomes without the ancestral A*03 allele (Table 4), favors the localization of such a putative trait between *HLA-A* and *PGBD1*. Future studies in these populations should consider selecting “non-conserved” chromosomes, i.e., those with discontinuous regions of conservation to the consensus haplotypes, to facilitate the search for individual loci contributing to the trait.

### Effect of the SNP Microhaplotypes on the Iron Overload Phenotype

In addition to its association with a “low CD8 phenotype”, the conservation of the ancestral A-A-T microhaplotype had been previously shown to be associated with a more severe iron overload phenotype [18]. In the present study we showed that, although in general, the presence in homozygosity of the A-A-T microhaplotype was associated to higher values of serum ferritin and total body iron stores in male patients, this association was not significantly sustained at the individual populations’ level and therefore cannot be used as a reliable universal marker of the phenotypic expression in HH (Table 5). The lack of statistical power in individual populations could be explained by the low numbers of non-A-A-T homozygous patients found in each region together with a high phenotypic diversity in these patients (reflected in the high range of values shown in Table 5), but it could also be influenced by genetic differences among populations, namely the loss of association of the A-A-T microhaplotype with the putative “low CD8 phenotype” marker in the Nord-Trøndelag patients. One should also note that these patients were mainly identified by screening of asymptomatic subjects, therefore unselected for clinical severity. On the contrary, Porto and Alabama, patients were mainly diagnosed on a clinical setting. Nevertheless, even using the same selection criteria, there are also great differences in iron loading between Porto and Alabama patients that could be related with different environmental factors or local life-style habits, including regular alcohol consumption. Therefore, further studies are still needed in larger populations and with a higher density mapping of the region, in order to find a more specific and universal surrogate marker of iron overload severity in HH.

### Concluding Remarks

We conclude that the evolutionary history of long extended haplotypes on chromosome 6p21.3 could account for heterogeneity within the haplotypes and consequent differences in the phenotypic expression of persons with *HFE* C282Y related HH. These observations have important implications for the interpretation of genotype/phenotype association studies in HH such as in the case of MHC loci associated with the transmission of the phenotype of low CD8^+^ T lymphocyte numbers where differences occur among HH populations from geographically distant regions (namely in north Portugal, Norway and Alabama, USA) or the association of MHC markers with iron overload. The effect of haplotype conservation may also have implications for understanding differences in disease penetrance or the consequences of patients’ sampling according to different detection methods. Although no consistent evidence is given about the predictive value of the A-A-T microhaplotype for individual purposes, one may predict that, in general, any cohort of severe or symptomatic HH patients may contain a high frequency of this conserved ancestral haplotype associated with a “low CD8 phenotype”, such as we have consistently found in Portuguese patients. On the contrary, in populations where programs for screening of asymptomatic cases are implemented, such as in Norway, it will be more probable to find HH patients with non-conserved haplotypes that are not associated with low CD8^+^ T lymphocytes. It remains unknown whether the severity of iron overload depends directly on the “low CD8 phenotype” or whether another independent modifier of the iron phenotype is inherited in linkage disequilibrium. Naturally, future positional cloning of a long-sought major genetic trait in MHC associated with the transmission of CD8^+^ T lymphocyte numbers [45] should provide answers to this question.

## Supporting Information

Table S1 **Comparison of the most common HLA allele, HLA A–B haplotype (uncorrected data) and SNP microhaplotype frequencies among three different populations of HH patients.**
(DOCX)
