# Supplementary material for: Effects of Highly Conserved Major Histocompatibility Complex (MHC) Extended Haplotypes on Iron and Low CD8+ T Lymphocyte Phenotypes in HFE C282Y Homozygous Hemochromatosis Patients from Three Geographically Distant Areas
Source: PLoS One. 2013 Nov 25;8(11):e79990. doi: 10.1371/journal.pone.0079990 (PMC3839968; doi:10.1371/journal.pone.0079990)
Supplement: Table S1 — Comparison of the most common HLA allele, HLA A–B haplotype (uncorrected data) and SNP microhaplotype frequencies among three different populations of HH patients. (DOCX) [file pone.0079990.s001.docx]

**Table S1 - Comparison of the most common HLA allele, HLA A-B haplotype (uncorrected data) and SNP microhaplotype frequencies among three different populations of HH patients.**

|  | **Porto**  **(n=130)** | **Alabama**  **(n=114)** | **Nord-Trøndelag**  **(n=364)** | ***P**** |
| --- | --- | --- | --- | --- |
| **HH chromosomes of patients from:** |  |  |  |  |
|  |  |  |  |  |
| **HLA- A alleles** | [%(n)] | [%(n)] | [%(n)] |  |
|  |  |  |  |  |
| **HLA-A*01** | 12.3 (16) | 14.9 (17) | 12.9 (47) | n.s. |
| **HLA-A*02** | 17.7 (23) | 15.8 (18) | 20.9 (76) | n.s. |
| **HLA-A*03** | 40.8 (53) | 47.4 (54) | 42.0 (153) | n.s. |
| **HLA-A*11** | 3.1 (4) | 3.5 (4) | 8.0 (29) | n.s. |
| **HLA-A*24** | 3.8 (5) | 3.5 (4) | 5.8 (21) | n.s. |
| **HLA-B alleles** | [%(n)] | [%(n)] | [%(n)] |  |
| **HLA-B*07** | 23.8 (31) | 30.7 (35) | 28.8 (105) | n.s. |
| **HLA-B*08** | 10.0 (13) | 11.4 (13) | 9.1 (33) | n.s. |
| **HLA-B*14** | 3.8 (5) | 9.6 (11) | 7.1 (26) | n.s. |
| **HLA-B*15** | 1.5 (2) | 1.8 (2) | 6.0 (22) | n.s. |
| **HLA-B*35** | 12.3 (16) | 3.5 (4) | 5.5 (20) | *.009*** |
| **HLA-B*40** | 7.7 (10) | 0.9 (1) | 9.6 (35) | *.008*** |
| **HLA-B*44** | 12.3 (16) | 14.0 (16) | 16.2 (59) | n.s. |
| **HLA Haplotypes** | [%(n)] | [%(n)] | [%(n)] |  |
| **A1-B8** | 5.4 (7) | 7.9 (9) | 7.7 (28) | n.s. |
| **A2-B44** | 3.1 (4) | 7.0 (8) | 6.3 (23) | n.s. |
| **A3-B7** | 16.9 (22) | 27.2 (31) | 21.4 (78) | n.s. |
| **A3-B14** | 1.5 (2) | 7.0 (8) | 6.0 (22) | n.s. |
| **SNP microhaplotypes** | [%(n)] | [%(n)] | [%(n)] |  |
| **A-A-T** | 90.8 (118) | 86.0 (98) | 76.5 (276) | 0.0003 |
| **G-G-G** | 6.2 (8) | 9.6 (11) | 16.0 (59) | 0.003 |
| **Non A-A-T nor G-G-G** | 3.1 (4) | 4.4 (5) | 7.5 (27) | n.s. |

*Comparisons among populations were done using the Chi-square test (*P* values indicated)

** not statistically significant after Bonferroni correction
